# The mzqLibrary – An open source Java library supporting the HUPO‐PSI quantitative proteomics standard

**DOI:** 10.1002/pmic.201400535

**Published:** 2015-07-14

**Authors:** Da Qi, Huaizhong Zhang, Jun Fan, Simon Perkins, Addolorata Pisconti, Deborah M. Simpson, Conrad Bessant, Simon Hubbard, Andrew R. Jones

**Affiliations:** ^1^ Institute of Integrative BiologyUniversity of LiverpoolLiverpoolUK; ^2^ The Faculty of Life SciencesUniversity of ManchesterManchesterUK; ^3^ The School of Biological and Chemical SciencesQueen Mary University of LondonLondonUK

**Keywords:** Bioinformatics, Data standard, MzQuantML, Proteomics standards initiative (PSI), Software, XML

## Abstract

The mzQuantML standard has been developed by the Proteomics Standards Initiative for capturing, archiving and exchanging quantitative proteomic data, derived from mass spectrometry. It is a rich XML‐based format, capable of representing data about two‐dimensional features from LC‐MS data, and peptides, proteins or groups of proteins that have been quantified from multiple samples. In this article we report the development of an open source Java‐based library of routines for mzQuantML, called the mzqLibrary, and associated software for visualising data called the mzqViewer. The mzqLibrary contains routines for mapping (peptide) identifications on quantified features, inference of protein (group)‐level quantification values from peptide‐level values, normalisation and basic statistics for differential expression. These routines can be accessed via the command line, via a Java programming interface access or a basic graphical user interface. The mzqLibrary also contains several file format converters, including import converters (to mzQuantML) from OpenMS, Progenesis LC‐MS and MaxQuant, and exporters (from mzQuantML) to other standards or useful formats (mzTab, HTML, csv). The mzqViewer contains in‐built routines for viewing the tables of data (about features, peptides or proteins), and connects to the R statistical library for more advanced plotting options. The mzqLibrary and mzqViewer packages are available from https://code.google.com/p/mzq‐lib/.

**Abbreviations**
API: application programming interface
CV: controlled vocabulary
PCA: principal component analysis
PSI: proteomics standards initiative
PSM: peptide‐spectrum match
XML: extensible markup language
XSD: XML schema definition

## 1 Introduction

The Proteomics Standards Initiative (PSI) is a collective of academic groups, journal editors and industrial organisations (mainly instrument or software vendors) working together to improve data sharing and reporting for proteomics. The PSI produces a number of output types as follows. First, the PSI has written minimum reporting guidelines describing what information should be included in a materials and methods section of an article or accompanying a data set, described in a “parent” document 1 and a set of modules describing individual techniques used in proteomics 2, 3, 4, 5, 6, 7, 8. Second, the PSI has developed standard data formats, including mzML for raw or processed MS data 9, mzIdentML for peptide or protein identification data 10 and two formats with different levels of support for quantitative data – mzQuantML 11 and mzTab 12 (further described below). Third, the different data formats require a common terminology set, which is captured in a controlled vocabulary (CV) – called the PSI‐MS CV 13. The PSI‐MS CV is updated approximately every week, and currently contains >2400 different entries (release 3.76.0, May 2015), including terminology for names, models/versions and parameters for instruments and software used in proteomic analysis, as well as various other necessary concepts. Each entry contains a term name, a definition, the unit that should accompany a given parameter if appropriate and synonyms etc. All of the formats described above must be populated with PSI‐MS CV terms to make valid examples (and which can be checked with validation software 14), thus ensuring that files produced from different exporters encode information in a consistent and systematic manner. Lastly, groups affiliated with the PSI produce software tools or programming interfaces to assist either end users or other bioinformatics developers to use the standards correctly.

The mzQuantML format (or “.mzq”) is XML‐based, and can contain a detailed trace of analysis steps that have been performed on data to detect and quantify 2D features in LC‐MS data, peptide‐level values inferred from these features, and protein or grouped protein (that cannot be separated due to shared peptides) quantitative values, in turn inferred by some methodology from peptide‐level values. The format is designed to store tables of quantitative data (so called *QuantLayers*, italic indicating element name in mzQuantML) for different levels (peptides, proteins or groups of proteins) derived from, and aligned across multiple samples (called *Assays*) or across pooled replicates (called *StudyVariables*), for example where some averaging process has been performed. mzQuantML can also store descriptions of the processing steps that have occurred at each stage in a pipeline, capturing the software name, parameters, inputs and outputs. Lastly, the format can store statistics (e.g. p‐values for differential expression across *StudyVariables*) or other scores associated with confidence of quantitative values. mzQuantML has several different modes – supporting individual types of technique: (i) MS^1^ label‐based (e.g. SILAC, N^15^, dimethyl labelling); (ii) MS^2^ tag‐based (e.g. iTRAQ or TMT); (iii) MS^1^ intensity‐based label free; (iv) label free spectral counting approach and (v) SRM 15. The same XML schema is shared across all modes, but the specific usage of general structures is governed by a set of “semantic rules” describing which structures should be used in each mode – which is checked by validation software 16. As such, mzQuantML reading software is able to determine the quantitation mode in a given file, supporting the capability for designing analysis algorithms for only a subset of all proteomic techniques.

There is a close association between mzQuantML and the recently released mzTab format 12. mzTab similarly captures quantitative values about peptides and proteins, measured across individual samples or grouped replicates (*Assays* and *StudyVariables*), but in a simplified tab‐separated format designed to be loaded straightforwardly into spreadsheet or statistical processing software. mzTab can also contain peptide and protein identification data, using simplified structures from the (more detailed) mzIdentML format. As such, mzTab is intended to be an output file from completed analysis pipelines, rather than acting as an internal analysis format supporting open source development.

We have previously developed a Java application programming interface (API) for mzQuantML, called jmzQuantML, which supports reading mzQuantML files into Java objects, and writing Java objects back to mzQuantML files on disk 16. In this article, we describe the development of new resources building on top of jmzQuantML – the mzqLibrary and the mzqViewer. The package is copyrighted under the Apache Software License 2.0 (http://www.apache.org/licenses/LICENSE‐2.0.html). In a label‐free quantitative proteomics pipeline (for example) there are a number of different steps that could be described as *pre‐processing/processing steps* performed by a variety of packages (reviewed in 17): e.g. baseline correction; feature detection and quantification; retention time (RT) alignment of chromatograms from parallel LC‐MS runs; identification of peptides and proteins for example by a search engine. There are then steps that could be described as *post‐processing –* mapping identifications onto features; inferring quantitative values for proteins or protein groups from peptide values; performing normalisation; performing statistics for differential expression testing 18, 19 and data visualisation. The mzqLibrary aims to provide open source mzQuantML‐supporting routines for post‐processing steps, i.e. all steps in a pipeline after feature detection and alignment of chromatograms (if performed). There are numerous examples of on‐going research to develop new algorithms for normalisation 20 and statistical testing 19, 21, 22, but new algorithms are often difficult to test in combination with data processed through standardised pipelines, due to file format incompatibilities. Similarly, although there are several excellent integrated proteomic pipelines and tools available, notably the TPP for example 23, we aim to provide tools that are fully compatible with the PSI standards. The provision of the mzqLibrary will therefore enable new routines developed by other groups to be plugged into a complete analysis pipeline, so long as their routine supports mzQuantML for input and output. We have also created a visualisation package, mzqViewer, which provides access to methods within the mzqLibrary and enables straightforward visualisation of mzQuantML files.

## 2 Materials and methods

### 2.1 Development of mzqLibrary and mzqViewer

The mzqLibrary and mzqViewer are both developed in Java and released with a permissive Apache 2.0 licence. The two packages both use jmzQuantML for file reading and writing, and embed several external libraries or tools for file format conversion, including jmzTab 24 and the progenesis post‐processor (PPP) 25. The mzqViewer uses JavaFX for several plots and visualisations, and calls out to R routines for principal component analysis (PCA) and heat map generation (http://www.r‐project.org/), which must be co‐installed by the user to gain full functionality of the mzqViewer. The prerequisites and installation guide for running mzqLibrary can be found at the website (https://code.google.com/p/mzq‐lib/).

### 2.2 Description of routines

The current release of both the mzqLibrary and mzqViewer is 1.0‐beta. The “beta” status reflects that a large number of different routines are contained, and all have been tested to a reasonable level of validation, but we expect that there will be some bugs reported as the software gains usage with mzQuantML files generated from a wider range of source pipelines. A 1.0‐final release will be made at the end of 2015. The following sub‐sections briefly describe each routine, the algorithms contained within, and the appropriate input and output formats – as summarised in Table 1. With the exception of the “VISUALISATION” routines (only available in the mzqViewer) – all other routines can be accessed via three different routes (i) command‐line access, which makes it possible to embed routines in other non‐Java based pipelines under any platform, e.g. Galaxy 26, (ii) Java API library for other Java applications to invoke routines directly and (iii) access from a simple graphical user interface (GUI) embedded in the mzqViewer – enabling end users to run single routines independently. We will later be releasing a full software package for proteomics analysis called ProteoSuite (http://www.proteosuite.org/), which will include the mzqLibrary, mzqViewer and quantitation by label‐free or tag‐based (iTRAQ/TMT) approaches.

**Table 1:** The routines presented in the current release of the mzqLibrary and mzqViewe

Type	Routine	Status	Techniques covered	Inputs (I:) and outputs (O:)	Parameters
IMPORTER	Progenesis LC‐MS converter	PPP Release 1.0.4	Label‐free	I: Peptide.csv or Feature.csv and Protein.csv (Progenesis) O: 1 mzq file	
IMPORTER	MaxQuant converter	1.0‐beta	Label‐free and SILAC	I: peptides.txt, proteinGroups.txt, summary.txt, experimentalDesignTemplate.txt O: 1 mzq file	None
IMPORTER	OpenMSconsensusXML converter	Alpha	Label‐free	I: 1 consensusXML file O: 1 mzq file containing quantified peptide list	None
EXPORTER	mzTab converter	1.0‐beta	All	I: 1 mzq O: 1 mzTab	None
EXPORTER	HTML converter	1.0‐beta	All	I: 1 mzq O: 1 HTML file	None
EXPORTER	CSV converter	1.0‐beta	All	I: 1 mzq O: 1 CSV (non‐standard format)	None
EXPORTER	XLS converter	1.0‐beta	All	I: 1 mzq O: 1 XLS (Excel) file	None
PROCESSING	IDMapper	1.0‐beta	Label‐free	I: 1 mzq file with feature lists and list of aligned but unidentified peptides and n mzIdentML files – one per FeatureList, O: 1 mzq file	The pairings of raw file name and mzIdentML file
PROCESSING	Normalisation	1.0‐beta	Label‐free	I: 1 mzq file containing “raw” peptide *QuantLayer* O: 1 mzq file containing “raw” and “normalised” peptide‐level *QuantLayers*	PSI‐MS CV accession identifying the data type of the input (raw peptide) *QuantLayer* and output (normalised peptide) *QuantLayer*
PROCESSING	Protein quant inference	1.0‐beta	Any that uses *AssayQuantLayers*	I: 1 mzq file containing quantified peptides O: 1 mzq file containing quantified peptides and protein groups	PSI‐MS CV accession identifying the data type of the input (peptide) *QuantLayer* and output (protein group) *QuantLayer*
PROCESSING	ANOVA	1.0‐beta	Any that uses *AssayQuantLayers* (no ratio‐based ANOVA)	I: 1 mzq file containing a protein or protein‐group level *AssayQuantLayer* O: 1 mzq file with added Global*QuantLayer* containing p‐values	N arrays of Assays; PSI‐MS CV accession identifying the data type of the input (protein or protein group) *QuantLayer*
VISUALISATION	Heat map	1.0‐beta	Any	I: 1 mzq file via mzqViewer; O: on‐screen or PDF	User selects the *QuantLayer*
VISUALISATION	Line plots	1.0‐beta	Any	I: 1 mzq file via mzqViewer; O: on‐screen	User selects peptides or proteins to plot
VISUALISATION	Principal Component Analysis	1.0‐beta	Any	I: 1 mzq file via mzqViewer; O: on‐screen or PDF	User selects the *QuantLayer*

#### 2.2.1 Progenesis LC‐MS converter

Progenesis LC‐MS (recently renamed to Progenesis QI from Waters Corp.) is a quantitative analysis software package for label‐free proteomics by LC‐MS. In the mzqLibrary, we have bundled the previously described PPP package from our group 25. Alongside other quantitation‐based functions, the PPP contains a converter for creating valid (label‐free) mzQuantML files from Progenesis results exported in the Peptide/Feature.csv and Protein.csv formats.

#### 2.2.2 MaxQuant converter

MaxQuant is popular free software for stable isotope‐based and label‐free quantitative proteomic analysis 27. We have included in the mzqLibrary a converter that takes as input MaxQuant files: evidence.txt, peptides.txt, proteinGroups.txt, ExperimentalDesignTemplate.txt and summary.txt, and converts to valid mzQuantML, supporting label‐free and SILAC workflows. The converter has been tested on the latest release of MaxQuant (version 1.4.0.8), but we cannot guarantee that the converter will function on all older and future releases of MaxQuant since the formats of the output files have not been stable across all releases.

#### 2.2.3 OpenMS consensusXML converter

OpenMS is flexible and powerful open source software for constructing analysis pipelines for proteomics 28. There is planned native support in OpenMS longer term for mzQuantML as an input and output format, but to date, there is no released converter for taking label‐free results in OpenMS and converting to mzQuantML. Hence, we have developed a simple converter for OpenMS consensusXML files (following alignment of parallel LC‐MS maps) and converting to mzQuantML files. Our converter is not a general purpose OpenMS to mzQuantML converter, and as such is flagged as an alpha release.

#### 2.2.4 mzTab, HTML, CSV and XLS converters

We have incorporated file format converters for exporting mzQuantML to various useful formats. The mzTab exporter embeds the jmzTab API 24, writing valid files that for example could be submitted to the ProteomeXchange public repository 29 or loaded into a spreadsheet or statistical software easily. To support end user visualisation of results and downstream analysis, we have also written exporters to a simple Comma Separated Values (CSV) file format (not‐standardised), equivalent version in XLS (Excel format) and HTML for viewing mzQuantML formatted results in web‐pages.

#### 2.2.5 IDMapper

Typically different algorithms or software packages used in analysis workflows for quantifying peptides (e.g. feature detection and quantification from MS^1^ data in LC‐MS maps) are from those used to identify peptides, via for example sequence database search of MS^2^ peak list spectra. Peptide (and protein) identifications and associated scores can be stored in the mzIdentML standard 10, and each peptide‐spectrum match (PSM) can be associated with the RT when the scan was generated. It is possible for statistics and other post‐processing steps on identification results to be performed using the mzidLibrary from our group 30. For quantification workflows there is a requirement to map identifications from PSMs to quantified features from MS^1^ data, which can be achieved by the IDMapper routine. The IDMapper can be used in scenarios for mapping search engine results (from any mzIdentML supporting search engine) onto quantified features from any software output that can be converted to mzQuantML – it is not used for the results presented in this manuscript, since identifications have already been mapped on features via Progenesis.

The routine is designed to take as input mzQuantML files that have *Feature* elements containing coordinates (*m/z* and RT) and peptide lists containing aligned (but as yet unidentified) *PeptideConsensus* elements (see 11 for a full description of elements and structures in mzQuantML). The routine requires *n* mzIdentML files as input – one per *FeatureList* (e.g. each sourced from one raw file analysed). If the software that generated the mzIdentML file did not include RT values for PSMs, then there is a routine in the mzidLibrary (*AddRetentionTimeToMzid*) for retrieving these values from the source spectrum file (currently supporting MGF formatted peak lists containing the attribute RTINSECONDS in the header of each spectrum). If the RT values are completely unknown, the IDMapper cannot function.

The IDMapper applies the following algorithm. In the first step, it loops through each of the *n* mzIdentML files – for which there will be *n* corresponding *FeatureLists* in the mzQuantML file. For each PSM, the *m/z* and RT are matched against each *Feature* in the appropriate *FeatureList*. Specifically, the *m/z* is tested for a match between the precursor *m/z* from the PSM (mzIdentML) versus the *m/z* of the quantified *Feature* (mzQuantML) within a given tolerance. The RT is tested whether it is within the reported RT window width of the *Feature*. If the mzQuantML file does not contain the RT window but only the RT centroid point, a standard tolerance window is applied (e.g. defaults to +/– 0.05 Da and +/– 10 sec). Meanwhile, users can also set their own tolerance window.

In the second step, the identifications are propagated up to the level of *PeptideConsensus* elements in mzQuantML. *PeptideConsensus* elements describe peptide/features that have been mapped as reporting on the same entity for multiple samples/replicates, for example in label‐free pipelines following RT alignment. The IDMapper populates *PeptideConsensus* elements with identifications (peptide sequence and modifications) if at least one *Feature* referenced from the *PeptideConsensus* has a mapped identification. In case of conflicts, i.e. the *PeptideConsensus* references Features in different *FeatureLists* carrying different peptide identifications, then the most common identification among the *FeatureLists* (largest number of *FeatureLists* carrying this identification) is assigned to the *PeptideConsensus*. Any conflicted identifications across *PeptideConsensus* elements are flagged in the file, using *userParam* elements.

#### 2.2.6 Normalisation

The normalisation method we have implemented as the reference method in the mzqLibrary follows the general scheme used in Progenesis LC‐MS (http://www.nonlinear.com) for label free global normalisation, based on the assumption that the majority of data points between a reference run and another run should be distributed around a log ratio = 0.

The routine takes parameters of: the level (peptide, protein or protein group); the type of input *QuantLayer* (currently only *AssayQuantLayer* supported in version 1.0‐beta, *RatioQuantLayer* to be supported in later releases); the CV accession and CV name identifying the data type of the input *QuantLayer*, e.g. MS:1001893: “Progenesis:peptide raw abundance”; optionally the identifier of a single *Assay* to be used as reference; a string used to identify any decoy peptides or proteins (so they are excluded from the input to normalisation); the CV accession and CV name to be assigned to the output *QuantLayer*, e.g. MS:1001891: “Progenesis: peptide normalised abundance”; and a *processingLevel*, indicating whether only identified peptides should be used for quantitation, or all peptides (all *peptideConsensus* elements identified and unidentified) – when performing peptide‐level normalisation.

The algorithm proceeds as follows. If no *Assay* is supplied as the reference run, then the procedure automatically determines the reference to use. This is achieved by using all *n Assays* independently as the reference run for normalisation (see below) – to produce *n* vectors of scaling factors, one for each possible reference. The algorithm calculates the standard deviation (stdev) of each vector, and then calculates the median stdev across all vectors. The chosen reference is that which has the stdev closest to the median stdev, to select a reference that is not an outlier and is central in terms of its position with respect to the others.

The normalisation procedure takes either the user‐defined reference *Assay* or the one automatically calculated, defined as *A_r_* here. For each non‐reference *Assay A_n_*, the following procedure is applied:

- Data is transformed onto a log ratio scale log_10_(*A*_r_ */A*_n_) for every peptide, protein or protein group value.
- Calculation of median absolute deviation (MAD). Assuming the data is normally distributed, the standard coefficient of 1.4826 is applied to the calculated MAD 31.
- Outliers, not within the 95% confidence interval (calculated as 2σ, where σ= 1.4826 MAD), are subsequently removed and steps (ii) and (iii) are repeated until convergence.
- Averaging (mean) of the remaining ratio values within the confidence interval is performed to derive a robust single estimate for each assay.
- Anti‐log transformation of the average yields the scaling factor for this given *Assay*.
- The scaling factors obtained from each Assay *A_n_* is multiplied by the data value for every row (peptide, protein or protein group) to create normalised values that are encoded within a newly created *AssayQuantLayer*.

Any “Null”, “NaN” or zero values for abundance are ignored for the purposes of the calculation of scaling factors, though retained subsequently.

#### 2.2.7 Protein Abundance Inference

We have included a routine for inferring protein group level quantification values from peptide‐level values, based on several mathematical operators. The routine requires input parameters to identify (by CV accession) the data types of input peptide *QuantLayers* (e.g. allowing for both raw and normalised *QuantLayers*), the parameters required to produce valid output in terms of CV accessions and CV term names for the new protein group level *QuantLayers*, and the mathematical operation to be performed (sum, mean, median). For label‐free quantification, the sum operation is strongly recommended.

The algorithm then performs the following steps. First, a mapping from peptides to proteins is obtained from the input file, and all peptides are classified as either “unique” (mapping to only a single protein accession), or “common” (mapping to same‐set or subset groups of proteins) 32. Peptides that are “conflicted” (mapping to multiple protein accessions that have unique peptides) are removed. To achieve this, protein groups are initially seeded from unique peptide‐protein mappings generated from the *ProteinList*, and expanded with same‐set proteins sharing common peptides. Secondly, protein group abundance is then calculated for both raw peptide abundance and normalised peptide abundance, respectively, using only the unique and common peptides; note, the latter will be unique to the protein group, but not necessarily the protein. A single group leader protein is then selected to represent each group (flagged with CV term “anchor protein”). This will be the single protein in a unique group, the first protein assigned to a same‐set group, or the protein associated with the most peptides in a sub‐set group. Protein abundance data is then calculated according to the user selected method (sum, mean, median of peptide abundance values) and output as a protein group list in the output mzQuantML file, which includes the protein group information and the *AssayQuantLayers* (e.g. protein group raw abundance and protein group normalised abundance). More advanced protocols for protein quantification inference (for example weighted averaging by different schemes) will be added at a later date. In the current version, “Null” and “NaN” values are replaced with zero values in the calculation of protein abundance from peptide abundance, since for the recommended (sum) operation these produce identical results. When support for ratio‐based protein aggregation is added, more customisable options will be added for the treatment of missing values.

#### 2.2.8 ANOVAPValue

The routine provides an ANOVA statistical test over two or more groups of *Assays* to test for evidence of differential expression. The *ANOVAPValue* routine calculates the p‐value for each protein or protein group and adds an extra *GlobalQuantLayer* (containing the p‐values) to the output mzQuantML file. The user (or code calling the routine) must specify how *Assays* are grouped by providing a listing of *Assay* ID values, separated by commas, and using semicolon to separate groups. The calling code must also specify a CV accession used to identify the *AssayQuantLayer* containing the quantitative values over which the ANOVA will be performed. If there is a “Null” or “NaN” value of protein abundance, the p‐value is set to “NaN”. Zeroes in protein abundance are replaced with 0.5 as the calculation of p‐value is based on log of abundance values, following standard statistical convention 33.

#### 2.2.9 Data visualisations in the mzqViewer

**Figure 1:**
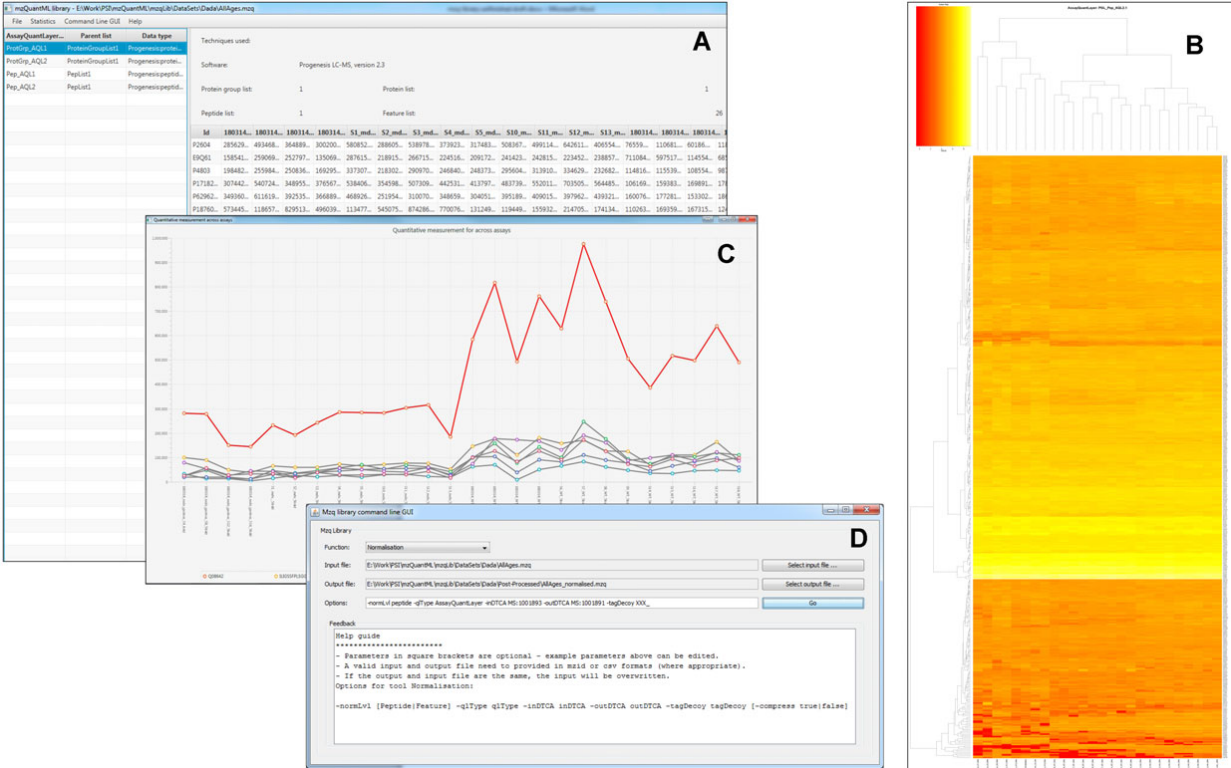
Screenshots of the mzqViewer. (A) The main window for viewing quantitative data within the file and calling other plotting features. (B) A heat map exported from the viewer in PDF format. (C) Line plots of a single protein and its constituent peptides. (D) The basic GUI for calling individual routines within the mzqLibrary.

The mzqViewer provides basic viewing functions for mzQuantML files. It displays the metadata of the file, including the quantitative technique (mode) used in the experiment, the software used to produce the data in the file, the detailed structure of the mzQuantML file (number of *FeatureLists*, *PeptideConsensusLists*, *ProteinLists* and *ProteinGroupLists*), and the table of all the *AssayQuantLayers* in the file. Users can browse the quantitation values in each *AssayQuantLayer* and display multiple selected rows in a line plot, for example to show how several proteins or peptides are behaving across different replicates or samples measured (Fig. 1). The mzqViewer also can call out to R to perform log scaling of all the data within any selected *AssayQuantLayer*, and generate a heat map which can be viewed in an R window, or saved as a PDF file. The software can also call out to R to generate a PCA on any *AssayQuantLayer* selected by the user (peptide or protein quantification values) to determine if individual Assays (for example within replicate groups) are clustering together.

### 2.3 Use cases for testing the mzqLibrary and mzqViewer

Case 1: for testing the routines present in the mzqLibrary, we have used data from an in‐house study on muscular dystrophy – the full biological interpretation of the results will be presented in a later manuscript. Briefly, muscular dystrophy is a family of genetic disorders characterised by progressive muscle loss, muscle fibrosis, chronic inflammation and premature death 34. Increased extracellular matrix (ECM) deposition is a hallmark of muscular dystrophy, however its exact composition and function in the regulation of fibrogenesis and myogenesis in dystrophic muscle are poorly understood. To explore the composition of the muscle ECM and identify ECM‐associated soluble factors, we developed a protocol to obtain protein fractions from mouse muscle enriched in extracellular and secreted proteins. We applied this protocol to compare the quantitative proteome profile from muscles of dystrophic mice (mdx) with muscles of wild type (wt) mice, using an LC‐MS label free workflow (see Supporting Information File 1 for methods) – processing the data using Progenesis LC‐MS. The results were exported directly from Progenesis in “Peptide.csv” and “Protein.csv” formats, specifying in the converter to only include results as raw abundance values at the peptide‐level in the produced mzQuantML file ‐ *mdx_progen_peptide_raw.mzq*, enabling us to test mzqLibrary routines for downstream processing. The source files and a batch file for reproducing our output is available from the project website: https://code.google.com/p/mzq‐lib/.

The following processing pipeline was applied to test the functionality of several routines of the mzqLibrary and mzqViewer. The *mdx_progen_peptide_raw.mzq* file was analysed by (i) mzqLibrary:ProteinAbundanceInference, (ii) mzqLibrary:Normalisation, (iii) mzqLibrary:ANOVA, (iv) export to mzTab and (v) various visualisations to check the data. The exported results were imported into the R statistical package (version 3.1.1) and compared versus the natively exported Progenesis Protein.csv file, for count of total protein groups quantified, Pearson correlation of log ratios (mdx/wt) and Pearson correlation of ANOVA p‐values.

Case 2: as a second test of the mzqLibrary on a sample with a controlled protein content, we analysed the CPTAC Study 6 data set – for details and methods see 35. In brief, the CPTAC set was created by splitting a single yeast culture into five equal samples (60 ng/uL yeast protein per sample), into which 48 human proteins were spiked at increasing concentrations (Sigma UPS1 standard at 0.25, 0.74, 2.2, 6.7 and 20 fmol/ul). These samples were designated A–E (A = lowest and E = highest concentration of spiked proteins) and run in triplicates on various MS platforms. The authors of the original study reported that UPS proteins could not be detected in the A samples, and thus we analysed B–E samples only. Comparisons of the spike‐in concentrations thus simulate “known” ratios, e.g. E/B (27:1); E/C (9:1); E/D (3:1) against a complex background assumed not to be changing. In previous analyses of this data set on various software packages, we have observed that the spike‐in UPS proteins are well controlled in the given ratios, but the yeast background is not optimally controlled at 1:1:1:1 in all conditions and thus the data set is not ideal for full benchmarking of differential expression analysis. Nevertheless, for the purposes intended in this article, i.e. to demonstrate comparable performance of the mzqLibrary reference methods and Progenesis for post‐processing, the data set serves as an adequate test case. We performed the same data processing steps (label‐free quantification in Progenesis; identification using Mascot – using the search database and parameters recommended in 35) and post‐processing steps as for Case 1. We additionally used R to generate boxplots of the log2 protein abundance values for mean E (three replicates)/mean B; mean E/mean C and mean E/mean D for the two pipelines.

## 3 Results and discussion

**Figure 2:**
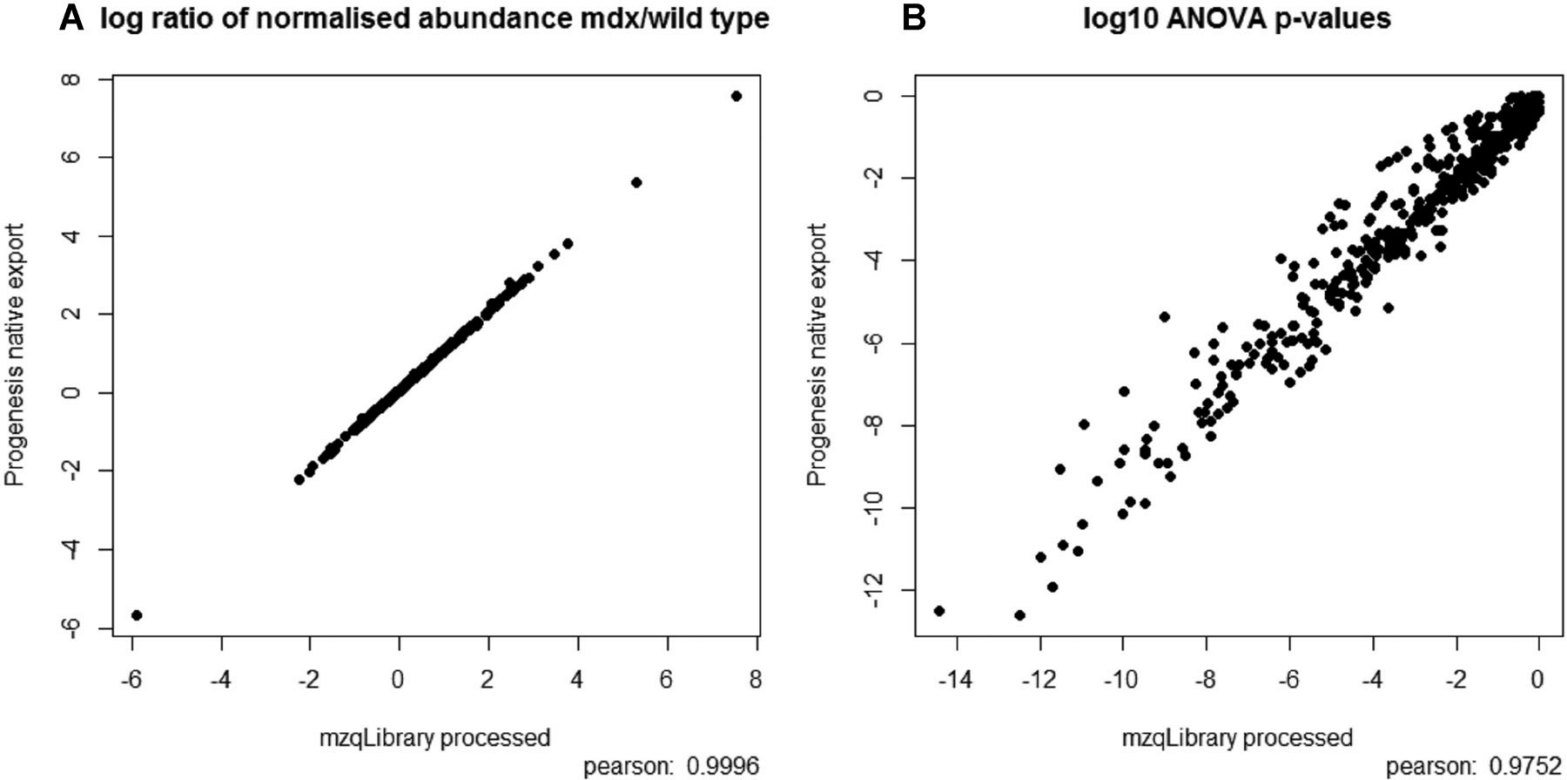
MDX processed data shown as (a) a scatter plot of log2 ratios (mean of mdx/mean of wt for normalised protein abundance) as produced natively by Progenesis and by mzqLibrary processing, and (b) as a scatter plot of log10(p‐values) as derived from an ANOVA test for differential expression on normalised protein abundance values, natively exported from Progenesis and from mzqLibrary processing.

To test the core function of routines within the mzqLibrary, we compared the results that are output by Progenesis LC‐MS natively against our own post‐processing of the same peptide quantification values exported from Progenesis into mzQuantML on the muscular dystrophy data set. We are thus demonstrating the following features – file format conversion from Progenesis to mzQuantML, using the mzqLibrary to perform peptide to protein quantification, normalisation and then ANOVA, using the same set of quantified peptides from Progenesis as the source. We wish to emphasise that we are not aiming to demonstrate that our modules are superior in performance to Progenesis, simply that they produce broadly the same results, as we are performing relatively standard types of routines. The modules are intended to act as reference methods, such that new developments in protein inference, normalisation and statistics can be compared against “standard practice”, potentially as part of a benchmarking study. This is an important point, since quantitative proteomics pipelines consist of multiple steps, and the mzqLibrary thus provides common routines to support fair comparisons of individual steps that would otherwise be impossible. It should be noted that for the protein inference step, due to the way in which data are exported from Progenesis, the method can only arrive at the same count of protein groups (Progenesis only exports peptides mapped to a single group leader). As expected, in the two pipelines, the same number of protein group‐level quantitation results were reported – 464 in both pipelines. We next performed a scatter plot and Pearson correlation on the log2 ratios of normalised protein abundance of the study groups (mdx/wt), as calculated by the mzqLibrary and exported natively from Progenesis as shown in Fig. 2(a). The majority of the ratios are identical (*r* ^2^ = 1), with very minor differences likely due to rounding errors. We next performed a scatter plot and Pearson correlations on the log10 p‐values (from ANOVA on normalised protein abundance across study groups) from both pipelines (Fig. 2(b)), which also displayed a high correlation *r* ^2^ = 0.97. The slight differences between the p‐values produced by the two pipelines are presumably due to differences in normalisation, and in particular, the method of selecting the reference run. It is known that ANOVA can be sensitive to alterations in normalisation, and as such, this is likely to explain the differences in p‐value observed. Nevertheless, using a cut‐off of p‐value <0.05 with Bonferroni correction (further dividing the threshold by the number of proteins identified 464), Progenesis classifies 121 proteins as differentially expressed and the mzqLibrary classifies 134 – with agreement on 118 of the proteins, indicating that differences in the two pipelines do not fundamentally alter the biological conclusions that would be drawn. We have also tested that the ANOVA routine produces identical results to those produced from Progenesis if provided with the same input data, e.g. protein group quantitation values converted directly from Progenesis (data not shown).

**Figure 3:**
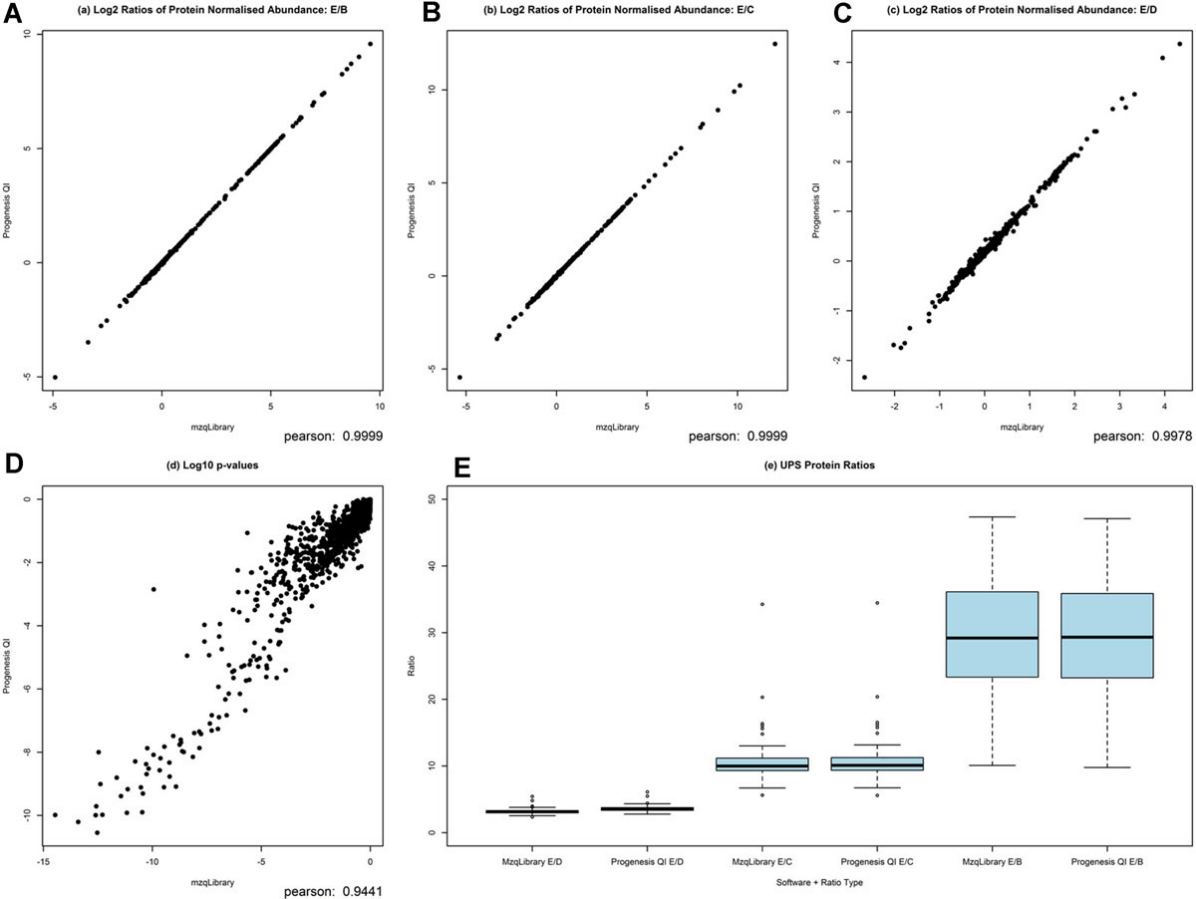
CPTAC processed data shown as a scatter plot of all protein log2 ratios for (a) E/B, (b) E/C and (c) E/D, showing a strong correlation between values derived from both Progenesis and the mzqLibrary. Log10 p‐values are shown for proteins (d) from the ANOVA test for differential expression, again indicating a good degree of agreement between values from Progenesis and the mzqLibrary. Finally boxplots are used (e) to show the ratios of UPS proteins for conditions B, C, D against E, from both Progenesis processed data and mzqLibrary processed data.

Full results from Case 2 (CPTAC data set) are presented in Fig. 3. We have made a comparison of log2 normalised protein abundance ratios. Figure 3(a–c) show the correlation of log2 normalised protein abundance ratios for conditions E/B, E/C and E/D (mean across replicates) respectively, between the mzqLibrary and Progenesis QI software packages. We also made comparison of log2 ANOVA p‐values between the mzqLibrary and Progenesis QI in Fig. 3(d). The boxplot in Fig. 3(e) shows normalised protein abundance ratios E/B, E/C, E/D for the mzqLibrary and Progenesis QI software packages, where the expected E/B ratio is 27:1, expected E/C ratio is 9:1, expected E/D ratio is 3:1. It demonstrates that both pipelines yield ratios that are highly similar and within the expected ranges. Table 2 shows the numbers of yeast and UPS proteins passing the significance threshold. In concordance with the results from Case 1, the results demonstrate highly similar results are obtained from post‐processing with either the mzqLibrary or Progenesis.

**Table 2:** Counts of proteins passing an ANOVA p‐value cut‐off of 0.05, with Bonferroni correction (dividing threshold by protein count of 1263) for various categories

Category	Protein count
Total proteins passing (ANOVA p‐value) threshold from mzqLibrary	109
Total proteins passing (ANOVA p‐value) threshold from Progenesis QI	81
Total proteins passing (ANOVA p‐value) threshold from both mzqLibrary & Progenesis QI	76
UPS proteins identified in both pipelines	47
UPS proteins passing (ANOVA p‐value) threshold from mzqLibrary	44
UPS proteins passing (ANOVA p‐value) threshold from Progenesis QI	44
UPS proteins passing (ANOVA p‐value) threshold from both mzqLibrary & Progenesis QI	44

The same 44 (out of a potential 47 identified) UPS proteins are identified as differentially expressed in mzqLibrary and Progenesis data sets. The stability of the yeast lysate background is unknown, thus the identifications of 76 yeast proteins as differentially expressed (by both packages – out of 81 classified by Progenesis QI for example) is not necessarily an indication of the quality of the analysis.

We have also profiled the memory and CPU requirements for analysis of case studies 1 and 2. Only three mzqLibrary routines were applied to our example cases (1 and 2) and their performance metrics are shown in Table 3.

**Table 3:** The performance metrics of the mzqLibrary in terms of speed and memory usage of each use case for three mzqLibrary routines (i.e. Normalisation, ProteinInference and ANOVAPValue)

	File size (MB)	Normalisation		ProteinInference		ANOVAPValue	
		Running time (second)	Memory (MB)	Running time (second)	Memory (MB)	Running time (second)	Memory (MB)
Use case 1	51.2	90	754	104	557	57	300
Use case 2	16.9	39	406	44	240	21	85

The “File size” is measured from the input mzq file before normalisation routine of each case. The “Running time” and “Memory” values are measured using JProfiler (version 8, https://www.ej‐technologies.com/products/jprofiler/overview.html) on a PC with Xeon® E5‐2630 v2 2.6GHz 2.6GHz CPU and 32GB RAM.

We plan to make further improvements and developments to the mzqLibrary to support open source developers. These developments include incorporating state‐of‐the‐art routines for protein inference, outlier rejection, normalisation and advanced/bespoke statistical tests, as well as support for more complex workflows, for example where pre‐fractionation of proteins has occurred. We welcome feedback on the library, and will endeavour to work with external groups to incorporate modules and libraries requested.

## 4 Concluding remarks

We anticipate that the release of the mzqLibrary will make it simpler for the following tasks to be performed. First, through the inclusion of converters from Progenesis LC‐MS (and Progenesis QI), MaxQuant and OpenMS, it will be more straightforward for bioinformatics groups to compare and benchmark multiple software packages for label‐free analysis, using the same methods (in the mzqLibrary) downstream of feature detection and alignment, thus removing variability in these steps. Second, the mzqLibrary should help bioinformatics groups wishing to develop new algorithms for individual analysis steps to construct complete pipelines to benchmark their tools. Third, the provision of the mzqViewer should help both bioinformaticians and lab‐based scientists to visualise and understand their data. Lastly, the two tools used in combination should help groups to prepare quantitative data in PSI standard format (mzQuantML or mzTab) for submission to PRIDE/ProteomeXchange 29 in support of published studies.

## Supporting information

As a service to our authors and readers, this journal provides supporting information supplied by the authors. Such materials are peer reviewed and may be re‐organized for online delivery, but are not copy‐edited or typeset. Technical support issues arising from supporting information (other than missing files) should be addressed to the authors.

Supplementary File 1 – Methods used in the LC‐MS analysis of dystrophic mice
