# Supplementary material for: The mzqLibrary – An open source Java library supporting the HUPO‐PSI quantitative proteomics standard
Source: Proteomics. 2015 Jul 14;15(18):3152–62. doi: 10.1002/pmic.201400535 (PMC4973685; doi:10.1002/pmic.201400535)
Supplement: Supplementary file 1 — – Methods used in the LC‐MS analysis of dystrophic mice [file PMIC-15-3152-s001.docx]

**Supplementary File 1 – Methods used in the LC-MS analysis of dystrophic mice**

***Muscle sample processing*:**

Mice were housed in a pathogen-free facility at the University of Liverpool in accordance with the Animals (Scientific Procedures) Act 1986 and the EU Directive 2010/63/EU and after local ethical review and approval by University of Liverpool’s Animal Welfare and Ethical Review Body (AWERB). Dystrophic (mdx4cv strain) and wild type (C57Bl/6 strain) mice where humanely sacrificed, the gastrocnemius muscles dissected and processed using a combination of gentle collagenase digestion and mechanical dissociation to release myofibre fascicles, which were then incubated in phosphate buffer saline (PBS) + 1% trypsin (Promega) for 40 minutes at 37°C. This procedure exposes the muscle endomysium and the myofibre basement membrane to trypsin and allows the release of protein fractions enriched in ECM components and ECM-bound factors.

A volume of sample equivalent to 50 μg of protein was diluted to a volume of 80 μL with 25 mM ambic. Samples were then age and genotype randomised and treated to achieve: protein denaturation, reduction, alkylation and trypsin digestion. To check for complete digestion each sample was analysed pre- and post-acidification by SDS-PAGE.

***High resolution LC-MSMS analysis:***

A 5-fold dilution of digest (100 ng protein equivalent) was injected on-column and chromatographed over a 50 min gradient using a method whereby following a survey scan at 70,000 resolution the top 10 most abundant peptide ions are fragmented and measured at high resolution (35,000) in the Orbitrap analyser to a mass accuracy of 0.01Da.

***LC separation:***

All peptide separations were carried out using an Ultimate 3000 nano system (Dionex/Thermo Fisher Scientific). For each analysis the sample was loaded onto a trap column (Acclaim PepMap 100, 2cm x 75μm inner diameter, C_18_, 3 μm, 100Å) at 5 μL/min with an aqueous solution containing 0.1% (v/v) TFA and 2% (v/v) acetonitrile. After 3 min, the trap column was set in-line with an analytical column (Easy-Spray PepMap® RSLC 15 cm x 75 μm inner diameter, C_18_, 2 μm, 100 Å (Dionex). Peptide elution was performed by applying a mixture of HPLC grade water with 0.1% (v/v) formic acid (solvent A), and HPLC grade acetonitrile 80% (v/v) with 0.1% (v/v) formic acid (solvent B). Separations were performed by applying a linear gradient of 3.8% to 50% solvent B over 30 min at 300 nL/min followed by a washing step (5 min at 99% solvent B) and an equilibration step (15 min at 3.8% solvent B).

***Q Exactive set-up:***

The Q Exactive instrument was operated in data dependent positive (ESI+) mode to automatically switch between full scan MS and MS/MS acquisition. Survey full scan MS spectra (*m/z* 300-2000) were acquired in the Orbitrap with 70,000 resolution (*m/*z 200) after accumulation of ions to 1x10^6^ target value based on predictive automatic gain control (AGC) values from the previous full scan. Dynamic exclusion was set to 20 s. The 10 most intense multiply charged ions (*z* ≥ 2) were sequentially isolated and fragmented in the octopole collision cell by higher energy collisional dissociation (HCD) with a fixed injection time of 120 ms and 35,000 resolution. Typical mass spectrometric conditions were as follows: spray voltage, 1.9kV, no sheath or auxiliary gas flow; heated capillary temperature, 250 °C; normalised HCD collision energy 30%. The MS/MS ion selection threshold was set to 1 x 10^4^ counts and a 2 *m/z* isolation width was set.

***Progenesis label-free quantification software:***

The data was processed with Progenesis (version 4 Nonlinear Dynamics, Newcastle upon Tyne, UK). Samples were automatically aligned according to retention time. Default peak picking parameters were applied and features with charges from 2^+^ to 7^+^ were retained. Database searching was performed using Mascot (Matrix Science, London, UK). A Mascot Generic File, created by Progenesis, was searched against the mouse UniProt database. A fixed carbamidomethyl modification for cysteine and variable oxidation modification for methionine were specified. A precursor mass tolerance of 10 ppm and a fragment ion mass tolerance of 0.01 Da were applied. The results were then filtered to obtain a peptide false discovery rate assigned to the Mascot search.
